# Adaptation and validation of the Instrumental Expressive Social Support Scale in Portuguese older individuals*

**DOI:** 10.1590/1518-8345.2647.3096

**Published:** 2018-11-29

**Authors:** Lígia Lima, Célia Santos, Celeste Bastos, Marina Guerra, Maria Manuela Martins, Patrício Costa

**Affiliations:** 1 Centro de Investigação em Tecnologias e Serviços de Saúde, Escola Superior de Enfermagem do Porto, Porto, Portugal.; 2 Universidade do Porto, Faculdade de Psicologia e Ciências da Educação, Porto, Portugal.; 3 Universidade do Minho, Escola de Medicina, Instituto das Ciências da Vida e da Saúde, Braga, Portugal.

**Keywords:** Validation Studies, Factor Analysis, Statistical, Social Support, Geriatric Nursing, Affect, Medication Adherence

## Abstract

**Objective:**

to adapt and validate the Instrumental Expressive Social Support Scale (IESS) in a sample of older people.

**Method:**

methodological study. The sample of 964 community-dwelling older people was randomly divided into two groups. The first group was used as a calibration sample to study the number of factors underlying social support through Principal Axis Factoring, and the second group as a validation sample to test the “best fit” model through Confirmatory Factor Analysis.

**Results:**

exploratory Factor Analysis suggested a three-factor solution, which was confirmed by Confirmatory Factor Analysis. The factors were similar to those in the pre-existing dimensions of the original instrument and were named as Sense of control (α = 0.900), Financial support (α = 0.802), Familiar and socio-affective support (α = 0.778). Confirmatory Factor Analysis showed acceptable fit. The model’s goodness-of-fit indexes were satisfactory (χ^2^/df = 5.418; CFI = 0.903; NFI = 0.884; RMSEA = 0.098). The convergent validity was supported by associations between social support and medication adherence and positive affect. The discriminant validity was evidenced by association with negative affect. The reliability analysis showed high values of internal consistency.

**Conclusion:**

the instrument proved to be a valid measure for the assessment of social support in older people.

## Introduction

Social support has been widely studied as a major determinant of health and well-being throughout the life cycle^(^ ^1^ ^-^ ^2^ ^)^ with significant importance in older age^(^ ^3^ ^)^.

Portugal is one of the European countries in which there is a high rate of progressive aging of the population mostly due to declining fertility and increased life expectancy^(^ ^4^ ^)^. Other problems arise in this period of life, such as the “growth of dependent, disabled people and people suffering from chronic diseases, experiencing family destructuring, changes in family patterns, coupled with the increasing isolation that affects older people and mobility problems, among others”^(^ ^5^ ^)^, that are frequently negatively associated with social support. In addition, the economic crisis has produced substantial negative impacts in Portugal over the last eight years. In fact, in a cross-sectional study conducted to compare social support of older people in seven European countries, Portugal showed the lowest score^(^ ^3^ ^)^. Therefore, it is crucial to develop instruments to support research on this new emerging reality, in particular, instruments that are capable of measuring social support in old people.

Most of the prevailing social support conceptualizations focus on resources provided by strong relationships, acting either as single contributors to a person wellbeing or as buffers against adverse events^(^ ^6^ ^)^, assuming that they are particularly important in coping with critical situations and life transitions as aging. A distinction is usually made between received and perceived support. The first is related to the tangible assistance provided by the social network and the second results from the subjective evaluation of the first one. Only the perceived support has been regarded as consistently linked to health^(^ ^7^ ^)^, and it is often described as a critical resource for dealing with stress^(^ ^8^ ^)^.

Social support is usually conceptualized as a multidimensional construct, which usually includes three dimensions: 1) Affective/emotional support, that includes a perception of being cared and understood by significant others, like friends and family; 2) Instrumental/financial support, such as having sufficient income to meet the personal needs; or 3) Informational support, namely providing knowledge and feedback that will help to accomplish individual goals^(^ ^9^ ^)^. Previously developed research found that the affective dimension is particularly important for the well-being and general health status of older adults^(^ ^9^ ^)^.

Social support is determined by socio-demographic variables, such as gender, marital status, age and socio-economic status, and the influence of each variable is often complex and they usually interact with other factors. A higher perceived support is associated with being a women or living with a partner^(^ ^3^ ^,^ ^10^ ^)^. A higher level of education was also found to be associated with positive social support^(^ ^10^ ^)^. Age is also determinant and the old-old were also found to report lower levels of social support from friends when compared to the young-old^(^ ^11^ ^)^.

The association between social support and health outcomes is well documented. Social support is important not only for promoting better mental health, but also for a good physical health, reducing mortality by 50%, independently of age, gender and other health conditions^(^ ^12^ ^)^. Low perceived social support was found to be associated with poor self-rated health in older women^(^ ^13^ ^)^. In its turn, the high support from family, friends and social groups are important predictors of disease outcomes, both in Hispanic and Caucasian samples^(^ ^14^ ^)^.

The association between social support and subjective well-being in older age has also been established^(^ ^15^ ^-^ ^18^ ^)^. Positive and negative affects are two of the three components of subjective well-being (SWB), which also includes life satisfaction. Existing evidence shows that social support is positively correlated with positive affect and inversely correlated with negative affect^(^ ^16^ ^)^. For example, in a study with older persons (mean age of 73) found that social support was associated with positive affect^(^ ^19^ ^)^. This same result was found in a study conducted in Australia, showing a positive association between social support and positive affect^(^ ^20^ ^)^. A strong positive association was also reported between life satisfaction and social support in a study involving a sample of community-dwelling older adults^(^ ^19^ ^)^.

A strong association was found between lack of social support and psychological distress in home-dwelling older adults^(^ ^21^ ^)^. Depression is relatively common in the elderly, and social support can act as a buffer, protecting them from negative affect^(^ ^22^ ^-^ ^23^ ^)^. When comparing the association between age and social support in different age groups, stronger associations with well-being were evidenced in older adults^(^ ^23^ ^)^.

A previous research has already established the association between social support and patient medication adherence, namely in old and chronically ill persons^(^ ^24^ ^)^. A former study has demonstrated that social support influences diabetes medication adherence and non-pharmacological treatment^(^ ^25^ ^)^. The relationship between social support and medication adherence is particularly significant in older people. In this age group, most people suffer from multiple chronic illnesses (e.g. hypertension, cholesterol, diabetes) and need to take several medications. Older adults are also the largest users of prescribed medication^(^ ^26^ ^)^.

Lack of social support of home-dwelling elderly persons was also suggested as contributing to medication nonadherence, and the prevalence of nonadherence was shown to be higher in individuals who lived in their own houses^(^ ^27^ ^)^. Additionally, other studies stress that the problem of medication nonadherence is increasingly high in those persons living alone in their own houses, with little support from family or friends^(^ ^12^ ^)^.

The assessment of social support needs to be carefully considered, depending on the type of research, as well as on the characteristics of the population under study. Most importantly, when addressing older adults, the instruments in use must focus on distinctive aspects of this age group, namely on their social roles, relationships and psychological development. More specifically, and considering that the autonomy of the elderly persons is usually replaced by increased dependency on their close relatives and friends, it is fundamental that the instrument clearly captures the affective dimension in perceived social support. Moreover, due to the reduced functionality and independence, it is also important to assess the way old adults perceive social support. This perceived social support is characterized either by attitudes of respect towards the autonomy of the dependent person or, in contrast, social support is perceived as a form of excessive control and lack of sense of empowerment, because “the perception of personal control plays a critical role in the health and well-being of an older person”^(^ ^28^ ^)^. Finally, the economic dimension is also important, since Portugal is a country in which older people are an economically deprived/vulnerable group, The Instrumental Expressive Social Support Scale (IESS) meets all these demands since it includes items that measure all these aspects of the perceived social support^(^ ^29^ ^)^.

The IESS scale was previously adapted to the Portuguese population and the results evidenced good psychometric properties^(^ ^29^ ^)^. Reliability was assessed through internal consistency and the Cronbach’s alpha was 0.83 for the total scale. Exploratory factor analysis indicated six factors accounting for 62.1% of the variance. The three factors that explained most of the variance observed were: Factor 1 - socio-affective support; Factor 2 - sense of control and Factor 3 - financial support. The IESS has also been used in a study with cardiac patients, in which a moderate negative correlation was found between social support and perceived stress^(^ ^30^ ^)^. The instrument was also used in a sample of patients with vertebra-medullar lesion and a negative association was found between social support and depression^(^ ^31^ ^)^.

The aim of this study was to validate the Portuguese version of the Instrumental Expressive Social Support Scale^(^ ^29^ ^)^ in older adults.

## Method

In this cross-sectional and observational study, a non-probabilistic and convenience sampling technique was used, whose subjects were recruited as part of a larger research project. Participants were 964 community-dwelling older people, aged between 64 and 99 years (M= 74.4, SD=7.0), 392 (39.6%) were male and 572 (57.7%) were female. Most were married (n=612; 61.8%), and 26.3% were widows (n=261). Primary school (4 years) educational level was found in 70% of the sample (n=696).

For validation purposes, the total sample was randomly divided into two different samples (EFA and CFA). An overview of the characteristics of the study participants is presented in Table 1.

**Table 1:** – Characteristics of the participants (n* = 964). Porto, PT, Portugal, 2016

	Subsample A – EFA^†^ (*n* = 500)		Subsample B – CFA^ǂ^ (*n*= 464)	
		
	*n*	%	*n*	%
Gender				
Male	207	41.4	185	39.9
Female	293	58.6	279	60.1
Age				
64-75	299	59.8	271	58.4
76-85	154	30.8	148	31.9
86-100	38	7.6	36	7.8
Missing	9	1.8	9	1.9
Marital status				
Single	21	4.2	23	5.0
Married	302	60.4	295	63.5
Divorced	29	5,8	12	2,6
Widow	134	26,8	122	26,3
Missing	14	2.8	12	2.6
Education				
No formal education	73	14.6	81	17.5
Primary school – 4 years	357	71.4	317	68.3
Primary School – 6 years	33	6.6	29	6.3
Middle school – 9 years	16	3.2	14	3.0
Secondary School -12 years	13	2.6	12	2.6
Post-secondary education	0	0	2	0.4
Bachelor	0	0	2	0.4
Degree	5	1.0	5	1.1
Doctoral	1	0.2	0	0.0
Missing	2	0.4	2	0.4
Occupation				
Active	9	1.8	13	2.8
Non-active	486	97.2	448	96.6
Missing	5	1.0	3	0.6

*n – number of participants; ^†^EFA – Exploratory Factor Analysis; ‡CFA – Confirmatory Factor Analysis

Several instruments were used. The Instrumental Expressive Social Support Scale has been previously adapted to Portuguese^(^ ^29^ ^)^. The IESS scale is a multidimensional measure of social support that includes 20 items grouped into three dimensions. A 5-point Likert scale was used to determine the frequency by which participants were bothered with the described issues in the last 6 months (1 – “always or almost always”; 2 – “many times”; 3 – “sometimes”; 4 – “rarely”; and 5 – “never”. The total score is calculated by the sum of the items scores and may vary between 20 and 100, with a higher total score reflecting a better perception of social support and absence of presented problems.

The Reported Adherence to Medication (RAM) Scale Portuguese version^(^ ^32^ ^)^ is used to assess the levels of medication adherence, which includes the frequency by which patients adjust or change the prescribed dosages. It measures the levels of agreement as “sometimes forgetting to take, or sometimes altering the medication dosage” and the perceived frequency of forgetting and altering the medication dosage. These items are rated on a 5-point Likert scale, with a total score ranging from 4 (very adherent) to 20 (non-adherent).

The Portuguese version of the Negative Affect Schedule (PANAS)^(^ ^33^ ^)^. The PANAS scale is used to assess the positive and negative affects during the previous 12 months. It includes 20 emotion descriptors, grouped into two subscales: positive emotions (Positive affect – PA), with 10 items (Cronbach alpha =0.87); and negative emotions (Negative affect – NA), with 10 items (Cronbach alpha =0.89). A 5-point Likert scale is used to rate each item, from 1 – “nothing or slightly” to 5 – “extremely”. In each subscale the items average is calculated (ranging between a minimum of 10 and a maximum of 50), in which higher scores show higher levels of positive or negative emotions, respectively.

The data relating to gender, age, marital status, educational attainment and occupation were also collected using a socio-demographic questionnaire.

This study is part of a larger research project named “*Viver mais com mais idade: do contexto familiar ao apoio institucional*”, implemented in a joint collaboration between the Escola Superior de Enfermagem do Porto (ESEP) and Vila Nova de Famalicão City Council. Approval was obtained from the Research Ethics Committee of CINTESIS, nº 244-14. All participants were informed about the study objectives and those who agreed to participate signed an informed consent form. The local authorities contacted all potential participants. A team of trained interviewers conducted the data collection, by either administering the instrument and interviewing the participants, or handing the questionnaire and asking the individuals to self-complete it.

For data analysis, the sample was randomly divided into two groups. Not all of the respondents answered every question and, consequently, the numbers included in the analysis showed some slight variations. The missing values were replaced by the mean score when the amount of missing values for each case was equal or smaller than five. The normality of the distribution of the response of the items, assessed through the item responses, was confirmed by the calculation of kurtosis and skewness, considering SK <3 and K <8 as reference values^(^ ^34^ ^)^.

The factorial structure of the IESS was tested with a holdout method for cross-validation, randomly dividing the full sample into two subsamples of 500 (Subsample A) and 464 (Subsample B) participants. The subsample A was used for the scale calibration. An Exploratory Factor Analysis was performed using a Principal Axis Factoring as extraction method (reflective model) of factors underlying social support. The Cronbach’s alpha was calculated to assess the reliability of each of the factors. Reliability was considered adequate when α≥0.70^(^ ^35^ ^)^. The subsample B was used for the scale validation and the model obtained in PAF was confirmed using CFA (ML method; tests of significance and goodness-of-fit measures: Chi-square, CFI, GFI, TLI, RMSEA and SMRS).

Concurrent validity and divergent validity were assessed by estimating the correlation between social support and medication adherence and positive and negative affects. Divergent validity with negative affect respectively (Pearson’s correlation analysis).

The SPSS package v20 (IBM SPSS Statistics) and the AMOS statistical package v21 were used for all statistical analysis.

## Results

The Principal Axis Factoring (PAF) method was used for a first exploratory data analysis (with oblimin rotation and without forcing the previous number of factors), aiming to understand how data were naturally grouped. From this analysis, items 4, 8, 9 and 12 were excluded due to their low communalities (lower than 0.30). Later, a second exploratory factor analysis was performed and the results showed that items were grouped into three factors and all items (excluding items 18, 19 and 20) were loaded into a single factor, with values above 0.30, as indicated in Table 2.

**Table 2:** – Results of the exploratory factor analysis of Subsample A. Porto, PT, Portugal, 2016

Item no.	Communalities		Factor	
		
		1	2	3
Item 5	0.559	0.849		
Item 3	0.463	0.770		
Item 6	0.562	0.740		
Item 13	0.565	0.696		
Item 15	0.547	0.658		
Item 2	0.534	0.582		
Item 19	0.614	0.536		0.339
Item 20	0.526	0.452		0.356
Item 14	0.412	0.367		
Item 7	0.624		0.924	
Item 11	0.618		0.862	
Item 1	0.403		0.450	
Item 17	0.540			0.843
Item 16	0.471			0.667
Item 10	0.463			0.490
Item 18	0.484	0.309		0.363

Extraction Method: Principal Axis Factoring; Rotation Method: Oblimin with Kaiser Normalization; Rotation converged in 8 iterations

The factors extracted were similar to three of the six pre-existing dimensions of the original instrument and were named as *Familiar and socio-affective support* (items 2, 3, 5, 6, 12, 13, 14, 15, 19 and 20), *Sense of control* (items 10, 16, 17 and 18) and *Financial support* (items 1, 7 and 11). Cronbach’s alpha was used to calculate the reliability for each of the factors and the following results were found: *Familiar and socio-affective support* = 0.778; *Sense of control* = 0.900; *Financial support* = 0.802.

The Confirmatory Factor Analysis (CFA) was used to test the model suggested by the EFA, which included three inter-correlated latent variables (F1 to F3) and 16 observable variables. All items loaded onto their proposed factors (Model 1). An analysis of the modification indices was conducted and the model was re-specified through correlation between errors from items 5 and 6, 3 and 5, and this modified model (Model 2) showed a better fit for the data^(^ ^34^ ^)^.

Considering that, theoretically, social support is a multidimensional construct and that, empirically, the factors showed strong correlations with each other, a second-order factor was extracted, which allowed to calculate a total score for the social support scale, thus producing a third model (Table 3).

**Table 3:** – Summary of the results of the CFA* for the 3 models and fit indices. Porto, PT, Portugal, 2016

	Χ^2^/df ^†^	CFI ^ǂ^	NFI^§^	RMSEA^||^	TLI^¶^
1st model	6.430	0.878	0.884	0.098	0.882
2nd model	5.418	0.903	0.884	0.098	0.882
3rd model	5.418	0.903	0.884	0.098	0.882

*CFA – Confirmatory Factor Analysis; ^†^Χ^2^/df – Chi-square test (degrees of freedom); ‡CFI – Comparative Fit Index; ^§^NFI – Normed Fit Index; ^||^RMSEA – Root Mean Square Error of Approximation; ^¶^TLI – Tucker-Lewis Index

**Figure 1:**
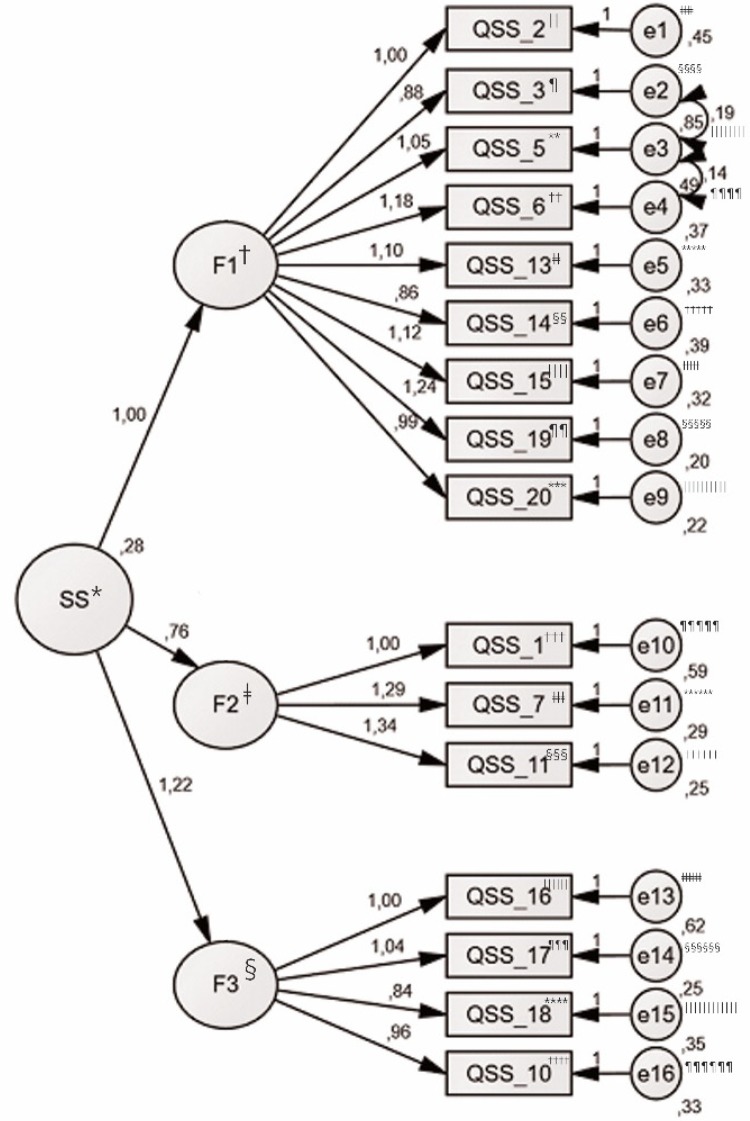
– Path diagram of the results of the CFA******* for the 3rd model

^*^SS – Social Support; ^†^F1 – Factor 1 (Familiar and socio-affective support); ^ǂ^F2 – Factor 2 (Financial support); ^§^F3 – Factor 3 (Sense of control); ^||^QSS_2 – Questionnaire of Social Support – item number 2; ^¶^QSS_3 – Questionnaire of Social Support – item number 3; ^**^QSS_5 – Questionnaire of Social Support – item number 5; ^††^QSS_6 – Questionnaire of Social Support – item number 6; ^‡‡^QSS_13 – Questionnaire of Social Support – item number 13; ^§§^QSS_14 – Questionnaire of Social Support – item number 14; ^||||^QSS_15 – Questionnaire of Social Support – item number 15; ^¶¶^QSS_19 – Questionnaire of Social Support – item number 19; ^***^QSS_20 – Questionnaire of Social Support – item number 20; ^†††^QSS_1 – Questionnaire of Social Support – item number 1; ^‡‡‡^QSS_7 – Questionnaire of Social Support – item number 7; ^§§§^QSS_11 – Questionnaire of Social Support – item number 11; ^||||||^QSS_16 – Questionnaire of Social Support – item number 16; ^¶¶¶^QSS_17 – Questionnaire of Social Support – item number 17; ^****^QSS_18 – Questionnaire of Social Support – item number 18; ^††††^QSS_10 – Questionnaire of Social Support – item number 10; ^‡‡‡‡^e1 – Error 1; ^§§§§^e2 – Error 2; ^||||||||^e3 – Error 3; ^¶¶¶¶^e4 – Error 4; ^*****^e5 – Error 5; ^†††††^e6 – Error 6; ^‡‡‡‡‡^e7 – Error 7; ^§§§§§^e8 – Error 8; ^||||||||||^e9 – Error 9; ^¶¶¶¶¶^e10 – Error 10; ^******^e11 – Error 11; ^††††††^e12 – Error 12; ^‡‡‡‡‡‡^e13 – Error 13; ^§§§§§§^e14 – Error 14; ^||||||||||||^e15 – Error 15; ^¶¶¶¶¶¶^e16 – Error 16; ^*******^CFA – Confirmatory Factor Analysis

The graphical expression of the path diagram, Figure 1, shows the factor loadings of the observed variables in the latent variables, as well as the co-variances between factors and variances of the items.

The psychometric sensitivity of the 16 items of the new version of the IESS scale was evaluated as measures of summary (mean, median, mode and standard deviation) and form (skewness and kurtosis) measures, presented in Table 4. The distributional properties and psychometric sensitivity were considered adequate when the absolute value of skewness was less than 3 and Kurtosis was less than 7, indicating a normal distribution of the responses to the items^(^ ^36^ ^)^.

**Table 4:** – Descriptive statistics of the items of the Social Support scale for Subsample B. Porto, PT, Portugal, 2016

ITEM NUMBER	Mean	Median	Mode	SD*	Skewness	Kurtosis
1	3.82	4.00	5	1.089	-0.638	0.969
2	4.15	4.00	5	0.912	-1.054	-0.311
3	3.73	4.00	4	1.073	-0.563	0.451
5	3.95	4.00	4	0.971	-0.862	0.325
6	4.10	4.00	5	0.950	-0.886	-0.562
7	3.42	3.00	3	1.132	-0.272	1.260
10	4.32	5.00	5	0.865	-1.248	-0.471
11	3.62	4.00	3	1.153	-0.511	1.088
13	4.22	4.00	5	0.893	-1.118	2.187
14	4.43	5.00	5	0.825	-1.511	1.451
15	4.29	5.00	5	0.896	-1.289	0.132
16	4.01	4.00	5	1.034	-0.872	0.725
17	4.15	4.00	4	0.859	-0.922	1.856
18	4.39	5.00	5	0.819	-1.374	0.174
19	4.18	4.00	5	0.887	-0.862	0.168
20	4.35	5.00	5	0.772	-0.924	0.969

*SD – Standard Deviation

The reliability of each of the three factors and the total score for sample B were calculated using Cronbach alpha coefficient and the following results were found: Familiar and socio-affective support (items 2, 3, 5, 6, 13, 14, 15, 19 and 20) = 0.911; Sense of control (items 10, 16, 17 and 18) = 0.805; Financial support (items 1, 7 and 11) = 0.866; Total score = 0.918. In this new version with 16 items, the scores range from 16 to 80.

The associations between social support and medication adherence and positive/negative affect were examined in order to test the convergent and divergent validity.

Very significant and positive associations were found between the total score of social support and adherence (r = 0.316; p = 0.000) and the positive affect (r = 0.216; p = 0.000), which supports the convergent validity of the IESS scale. The divergent validity of the IESS was established through the association between social support and negative affect, since a very significant negative correlation was also found between the total score of social support and negative affect (r = -0.371; p = 0.000).

## Discussion

The main aim of this study was to adapt and validate the Portuguese version of the Instrumental Expressive Social Support (IESS) scale in older adults.

An exploratory factor analysis was first conducted in the calibration sample to explore the number of factors underlying the social support measured by this scale, and the Principal Axis Factoring (PAF) was used to explore how items naturally clustered. The first analysis revealed that some modifications were required to improve the factor structure. This involved the exclusion of four items, namely items 4, 8, 9 and 12 due to the low factor loadings found. Items should not be excluded purely for statistical reasons, but after content analysis, and the exclusion was also acceptable for conceptual/theoretical reasons, since these items did not reflect distinctive aspects of social roles, relationships or social representations of the older adults about old age. Two items described the perceptions of having a less gratifying intimacy and sexuality and experiencing unhappiness with the marital status. In what concerns the first item, although literature suggests that intimacy and sexuality are important areas of personal gratification in all ages, evidence also shows that older people tend to value intimacy (that is, opportunities for companionship and love) more than physical contact/sexuality^(^ ^37^ ^)^. A research also stresses that there are prejudices about sexuality in old age^(^ ^38^ ^)^ and this could also explain why the item was not considered adequate in this population. Older people usually share social representations in which sexuality is seen as absent, unnecessary or inappropriate in their age group^(^ ^38^ ^)^. Perhaps because this type of social representations is still influential/present, old adults do not consider sexuality as an important component of social support. The item related to satisfaction with the marital status was excluded probably for similar reasons, as social expectations dictate that older people are not expected to change their marital status, for example, through divorce or marriage. The two remaining items could be considered unappropriated from a social or developmental standpoint, since they described the experience of having problems related to children and having a less satisfying job. The majority of participants were already retired and lived alone or with a partner and, as expected at this age, without children under their responsibility, so they did not share their daily life with children.

The reduced version was again analysed by PAF and three factors were extracted, which were similar to those of the pre-existing dimensions in the previous Portuguese version of the instrument. The three factors showed good reliability and were named as Familiar and socio-affective support, Sense of control and Financial support. Some items loaded in more than one factor, but all were grouped into the factor where their loading was higher.

The first factor, named “Familiar and socio-affective support”, groups the items that measure what the expressive dimension of social support usually describes. This dimension evaluates whether respondents feel or believe that their family and friends are close and affectionate, and that they are available for sharing their problems. It has been argued that close relatives and friends have different roles in providing social support in old age, but they both represent important sources of love and affection, and contribute to subjective well-being^(^ ^18^ ^)^. The second factor, “Financial support”, represents what is usually described as instrumental support, since it assesses if older people feel that they have sufficient financial support for their needs and if they feel able to manage their finances. As previously stated, this dimension is particularly relevant for Portuguese old adults, as they are a significant part of an economically deprived group^(^ ^3^ ^)^. Finally, the third factor, “Sense of control”, includes items that evaluate how respondents feel that their close relationships are capable of respecting their autonomy and independence by providing support that is not over controlling. A review of the literature showed that older people have a strong inner drive towards autonomous decision-making, despite the dependency^(^ ^28^ ^)^.

Subsample B was used for the scale validation and to confirm the 3-factor structure of the IESS scale, in order to show its usefulness in assessing social support in older adults. To the best of our knowledge, this is the first study conducted in order to examine the factor structure of the IESS scale and previous research papers only reported the exploratory analysis in the study of the psychometric properties of the instrument^(^ ^29^ ^)^. The inclusion of 2 correlations between errors in the model was necessary, but the results obtained by CFA suggest that the 3-factor model structure performed the best on the goodness-of-fit indices, similar to the consensus cut-offs^(^ ^35^ ^)^. A final step of CFA allowed extracting a second order factor that supports the existence of a total score for social support as measured by the IESS scale.

As previously argued, theory and evidence advocate that social support consists of multidimensional construct that can be assessed through certain dimensions or underlying sub-constructs that can be measured using a questionnaire with a certain number of items. The intercorrelations found between the three factors were also statistically significant, and sustain the existence of a main construct of social support as suggested in previous studies^(^ ^30^ ^-^ ^31^ ^)^.

The values for the dimensional and total internal consistencies were all at robust levels and higher than the values previously reported using the original version of the IESS scale^(^ ^29^ ^)^. In addition, the analysis of the psychometric sensitivity of each item revealed that the 16 items were all sensitive.

Concurrent validity and divergent validity were assessed by estimating the correlation between the IESS scale and medication adherence and the positive and negative affects respectively (Pearson’s correlation analysis). The analysis of the association with these other psychological constructs sustained the convergent validity and the divergent validity of the IESS scale, as it negatively relates with negative affect, in line with previous studies^(^ ^23^ ^,^ ^39^ ^)^, and positively relates with positive affect, also consistent with previous research^(^ ^16^ ^,^ ^18^ ^,^ ^39^ ^)^. The observed association between social support and medication adherence was also found in other studies^(^ ^24^ ^,^ ^40^ ^)^. With growing age and multimorbidity, medication regimens become increasingly demanding and it is expected that those who perceive high levels of social support are also those who have more resources to adhere to medication.

## Conclusion

Social support plays an important role in the health and well-being of older persons. This is the first study aimed at validating the Instrumental Expressive Social Support Scale (IESS) in Portuguese older people.

This study gives noteworthy contributions as it includes a large community-based sample, which in addition to providing a good and trustworthy analysis, also enables the generalization of findings outside the clinical contexts in which the IESS was previously used.

Finally, the IESS scale shows appropriate validity and good internal consistency and can be considered a useful instrument to measure the perceived social support in older people, enabling the identification of the most vulnerable areas and those that need further nursing interventions.

The present findings have important implications for clinical practice, since older people who perceive lower levels of social support were found to be more vulnerable to show negative affect and behaviours of medication nonadherence. The identification of these persons enables nurses to directly intervene as a supportive resource in promoting self-care and well-being for older people. The findings will likely contribute to the education and training of professional nurses and nursing students involved in the process of caring for older people. Additionally, the use of the IESS can be broadly extended to aged care settings to support future research.
